# Different EEG brain activity in right and left handers during visually induced self-motion perception

**DOI:** 10.1007/s00415-020-09915-z

**Published:** 2020-05-27

**Authors:** Michaela McAssey, James Dowsett, Valerie Kirsch, Thomas Brandt, Marianne Dieterich

**Affiliations:** 1 Department of Neurology, University Hospital, Ludwig-Maximilians-Universität München, Marchioninistraße 15, 81377 Munich, Germany; 2 German Center for Vertigo and Balance Disorders (DSGZ), University Hospital, Ludwig-Maximilians-Universität, Munich, Germany; 3 Graduate School of Systemic Neuroscience (GSN), Ludwig-Maximilians-Universität, Munich, Germany; 4 RTG 2175, Perception in Context and its Neural Basis, Ludwig-Maximilians-Universität, Munich, Germany; 5 SyNergy, Munich Cluster of Systems Neurology, Munich, Germany

**Keywords:** Roll vection, Self-motion perception, Visual motion perception, Thalamo-cortical vestibular dominance, Hemispherical lateralization, Right and left handers, EEG

## Abstract

Visually induced self-motion perception (vection) relies on visual–vestibular interaction. Imaging studies using vestibular stimulation have revealed a vestibular thalamo-cortical dominance in the right hemisphere in right handers and the left hemisphere in left handers. We investigated if the behavioural characteristics and neural correlates of vection differ between healthy left and right-handed individuals. 64-channel EEG was recorded while 25 right handers and 25 left handers were exposed to vection-compatible roll motion (coherent motion) and a matched, control condition (incoherent motion). Behavioural characteristics, i.e. vection presence, onset latency, duration and subjective strength, were also recorded. The behavioural characteristics of vection did not differ between left and right handers (all *p* > 0.05). Fast Fourier Transform (FFT) analysis revealed significant decreases in alpha power during vection–compatible roll motion (*p* < 0.05). The topography of this decrease was handedness-dependent, with left handers showing a left lateralized centro-parietal decrease and right handers showing a bilateral midline centro-parietal decrease. Further time–frequency analysis, time locked to vection onset, revealed a comparable decrease in alpha power around vection onset and a relative increase in alpha power during ongoing vection, for left and right handers. No effects were observed in theta and beta bands. Left and right-handed individuals show vection-related alpha power decreases at different topographical regions, possibly related to the influence of handedness-dependent vestibular dominance in the visual–vestibular interaction that facilitates visual self-motion perception. Despite this difference in where vection-related activity is observed, left and right handers demonstrate comparable perception and underlying alpha band changes during vection.

## Introduction

Self-motion perception relies on the contributions of multiple sensory systems, with the most important contributions from the visual and vestibular systems. Although vestibular stimuli invariably signal the sensation of self-motion, visual motion stimuli can produce two alternate interpretations with the observer perceiving that (a) they are stationary in a moving surround, i.e. object motion or (b) they are moving in a stationary surround, i.e. self-motion. When a stationary observer is presented with a large-field visual motion stimulation, a sensation of apparent self-motion, i.e. vection, occurs [1]. Vection highlights the important role of the visual system in self-motion perception. Indeed, while the vestibular system elicits information about body motion at acceleration and deceleration, it is visual information that allows us to perceive self-motion at a constant velocity (e.g. car motion).

Early positron emission tomography (PET) and functional MRI (fMRI) imaging studies have shown that optokinetic stimuli used to induce vection are associated with both activation of visual cortex and concurrent deactivation of parieto-insular vestibular cortex, PIVC [2–5]. These findings have been hypothesized to reflect an inhibitory reciprocal visual–vestibular interaction as a mechanism for self-motion perception, in which the dominant sensorial weight is shifted from one modality to the other more reliable modality [2, 6]. In terms of vection, inhibition of the vestibular cortex reflects the actual, missing vestibular input as compared to the expected vestibular input. It is important to note that the relationship between these systems is multifaceted and the observation of concurrent activation/deactivation of these systems is not itself indicative of vection presence. For example, visual motion stimulation has been shown to produce increased activity in the visual cortex and concomitant decreased activity in the PIVC even in the absence of vection [3, 7]. Furthermore, it has been demonstrated that the presence of acceleration in visual motion stimuli may lead to increased, rather than decreased, activations of the PIVC during vection [8, 9]. This modulation of the visual–vestibular interaction during visual self-motion is thought to result from selectivity of the PIVC to visual gravitational motion [10].

Distinguishing between activity that corresponds to visual motion stimulation versus that which corresponds to vection per se is a major challenge if we are to understand the neural basis of visual self-motion perception. Early studies identified large networks of regions with activations and deactivations attributed to visual self-motion perception [4, 11–14]. However, some of these studies did not directly test whether vection was experienced by participants, but rather relied on stimuli consistent with self-motion [14] or stimuli which were assumed to induce vection [11, 14], making it difficult to infer if the findings relate to visual motion stimulation or vection. Isolating vection-related neural activity is also made difficult by the fact that visual motion stimuli do not generate a continual sensation of vection, but rather tend to produce bistable perception with alternating periods of object- and self-motion perception [1, 5, 15]. Furthermore, even when vection is present, there are large differences in reported vection strength and duration between individuals [16].

The present study investigated the behavioural characteristics and neural correlates of vection in left and right handers because several PET and fMRI studies using vestibular stimulation have revealed a vestibular thalamo-cortical dominance in the right hemisphere in right handers and the left hemisphere in left handers [17–28]. EEG recordings synchronized with participants’ perceptual states were obtained in order to accurately distinguish between periods of object- and self-motion. Given that alpha oscillations have been linked to vection [29–31] and associated with bistable perception [32–34], the EEG analyses focused primarily on alpha band activity and on potential differences in this band between left and right handers.

## Materials and methods

### Participants

A total of 25 right handed (14 females, age: 27.68 years *SD*: 4.02 years) and 25 left handed (18 females, age: 24.83 years *SD*: 4.24 years) healthy adults participated in the experiment. All participants had normal or corrected-to-normal vision and reported no prior history of vestibular symptoms or neurological disorders. Participants completed the 10-item Edinburgh Handedness Inventory to determine their handedness (right handers: 8% >  + 40, 16% >  + 60, 16% >  + 70, 8% >  + 80, 52% >  + 90; left handers: 8% > − 40, 4% > − 50, 24% > − 60, 20% > − 70, 20% > − 80, 24% > − 90). The experiment was conducted in accordance with the guidelines of the institutional ethics committee and the Declaration of Helsinki. All participants gave their informed written consent prior to their participation and received compensation (€10/h).

### Visual motion stimulation

The stimuli comprised two movies: a coherent and an incoherent rotating pattern of dots. The stimuli were generated in MATLAB (The MathWorks Inc., Natick, MA, USA) using the Psychophysics Toolbox extensions. The stimuli consisted of 1000 randomly spaced white dots on a black background. A central green dot provided a fixation point. The dots rotated in the roll plane in either a clockwise (CW) or counter-clockwise (CCW) direction at 30°/s. In the coherent condition the dots rotated in a smooth circular formation. In contrast, the dots in the incoherent condition had a random sinusoidal movement in both the *X* and *Y* direction added to the overall circular trajectory (i.e. the phase and amplitude of the additional sinusoidal movement was randomized separately for each dot). This resulted in each individual dot appearing to follow a random trajectory, but with the global pattern maintaining either a CW or CCW direction with a mean velocity of 30°/s. The stimuli were projected onto a custom-built dome (diameter: 75 cm) apparatus with the rotation axis passing through the dome centre. The distance between the apex of the dome and the participant’s nasion was 31 cm, with the visual field subtending a visual angle of 100°. The experiment was conducted in a dark room with the visual stimulus covering the participant’s entire field of view, ensuring that no horizontal or vertical cues were observable.

### Experimental procedure

**Fig. 1:**
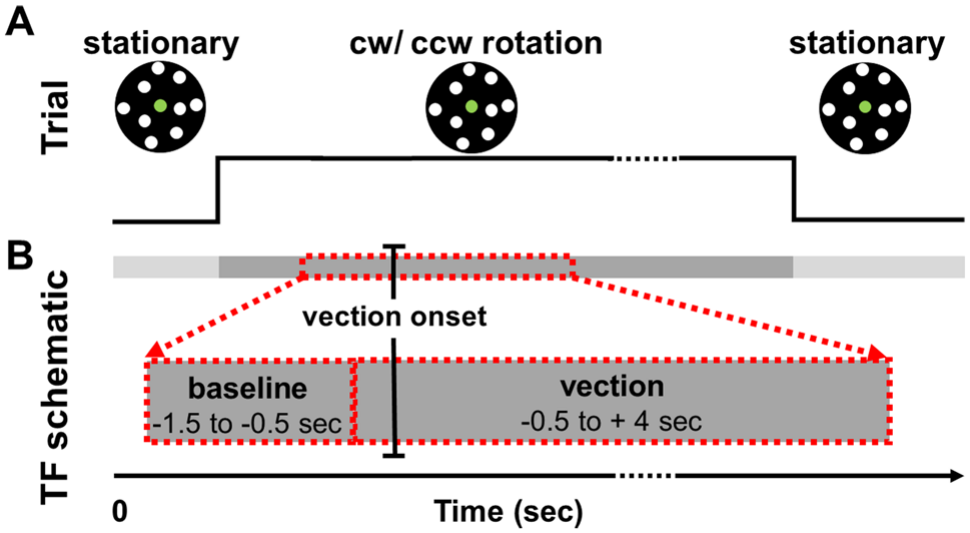
**a** Trial schematic. Each trial began with stationary dots for a jittered period of between 3 and 5 s. The dots then began to rotate in a clockwise (CW) or counter-clockwise (CCW) direction for 20 s. During this time the participants reported vection onset/offset by means of a button push. The dots then became stationary for a period of 10 s. Participants verbally reported the strength of their vection experience after each trial. **b** Time–frequency (TF) schematic. The data for the TF analysis were extracted from the 20 s period of dot rotation in the coherent condition. The baseline window (− 1.5 to − 0.5 s) and the ongoing vection window (− 0.5 to + 4 s) were defined relative to reported vection onset (time 0)

Prior to the beginning of the experiment, the height of the dome apparatus was adjusted such that participant’s line of sight was in-line with the centre of the dome. As illustrated in Fig. 1a each trial began with the presentation of stationary dots for a jittered period (range 3–5 s), followed by dot rotation (20 s) and then a return to stationary dots (10 s). Participants were instructed maintain fixation on the central green dot for the duration of the trial and to avoid following the moving dots with their eyes. During the dot rotation, participants reported vection onset and vection offset by means of a button push on a gaming controller. Separate buttons were denoted for perceived CW and perceived CCW vection onset/offset, with participants holding the controller with both hands and making responses with both index and middle fingers. At the end of each trial, participants were asked to verbally report on the strength of their vection experience on a scale of 0 (‘no vection’) to 10 (‘I felt I was really moving’). This response was recorded by the experimenter before the next trial begun. Participants were seated with their head on a chin rest during each trial. The experiment comprised a total of 100 trials: 50 coherent trials and 50 incoherent trials, each with 25 trials in CW and CCW directions. Trials were presented in a randomized order in blocks of 10 trials. Participants could take a self-timed break at the end of each block and between trials if necessary. Each session began with a short practice block (12 trials in a randomized order: 6 in each condition, with 3 in each direction) during which the participants could become familiar with the experimental procedure and self-calibrate their use of the vection strength scale. Following this practice, the EEG was prepared and the main experiment was conducted. No participant reported motion sickness and all participants completed the experiment.

### EEG acquisition and preprocessing

The EEG was recorded using a 64 active electrode system (EASYCAP and BrainProducts, GmbH, Germany). The electrodes were placed according to the international 10–10 system with the reference electrode placed at FCz. Horizontal and vertical eye movements were recorded using bipolar electrode montages. The data were collected at a sampling rate of 1000 Hz, with no additional online filters. The active electrode impedances were kept below 5 kΩ throughout the recording. The EEG recording was synchronized with the visual motion stimulation and response controller using triggers sent via parallel port to the EEG recording. The EEG data were acquired using BrainVision Recorder software (BrainProducts, GmbH, Germany) and subsequently analysed using EEGLAB [35], custom scripts in MATLAB and FOOOF [36] in Python. The data preprocessing was performed in the following order. The data were re-referenced to the common average reference. Independent component analysis (ICA) was then used to decompose the data using the runICA function in EEGLAB. Components reflecting ocular noise, such as blinks or muscle artifacts were removed and the EEG signals were then reconstructed.

### Fast Fourier transform (FFT) analysis

In order to compare the neural activity in the coherent and incoherent conditions, the data from motion onset to motion offset (i.e. 20 s of stimulus rotation data) was extracted for each participant. The CW and CCW trials were collapsed, resulting in 50 coherent and 50 incoherent trials per participant. Next, the 20 s of data in each trial was segmented in non-overlapping 1 s segments. In order to reduce the potential contribution of motor response artifacts or residual eye artifacts, any segment containing a button–push or channel signal exceeding 100 μV was rejected. The remaining segments were then multiplied with a Hanning window and spectra were computed using an FFT approach. A grand average was calculated for each participant, reflecting their respective spectral amplitude at each electrode position. Each participant’s individual peak in the theta (4–7 Hz), alpha (7–14 Hz) and beta (20–30 Hz) bands were then identified using the FOOOF algorithm [36], with the peak being defined as the largest fitted peak, within the respective band, above the 1/f component of the spectrum. This resulted in an individual peak, i.e. in each frequency band, at each electrode in both the coherent and incoherent conditions for each participant.

### Time–frequency (TF) analysis

In order to study the temporal dynamics of the alpha band activity at vection onset and during ongoing vection, a time–frequency (TF) decomposition was conducted on the coherent condition data. To be included in this analysis a trial had to (a) have at least 3 s between motion onset and vection onset and (b) have at least 5 s of continual ongoing vection after vection onset. For all trials meeting this criteria an 8 s window of data was extracted, ranging from 3 s before vection onset to 5 s after vection onset (i.e.  − 3 s to + 5 s, with time 0 = vection onset). A Morlet wavelet transformation was used to calculate TF maps, linearly ranging from 3 to 40 Hz, for each participant across all segments. The wavelet had a width of 7 cycles. The TF maps were normalized for each participant with respect to a pre-vection interval (− 2.5 s to − 1.5 s relative to vection onset) using a decibel conversion. Next, regions of interest (ROIs) were defined separately for left and right handers based on the results of the FFT analysis. The left hander ROI consisted of electrodes CP1, P3, CP3, P1, P5, PO7 and PO3, while the right hander ROI consisted of electrodes Pz, CP2, P1, P2 and CPz. The spectral power in the alpha band was then averaged within the respective ROIs. The baseline window was defined as − 1.5 s to − 0.5 s (Fig. 1b) and the time points in this window were averaged, resulting in one value per participant. The vection window was defined as − 0.5 s to + 4 s (Fig. 1b), with each participant having one value per time point within this range.

### Source localization

The exact low-resolution brain electromagnetic tomography algorithm (eLORETA), as developed and implemented by Pascual-Marqui [37, 38] and freely available through the LORETA webpage (https://www.uzh.ch/keyinst/loreta.htm), was used to estimate the location of EEG sources. The employed LORETA implementation uses a realistic head model [39] and restricts estimated solutions to cortical grey matter which is modelled by 6239 voxels of 5 mm resolution. The LORETA analysis included coherent condition trials in which vection was present and incoherent trials in which vection was absent. For coherent trials, each participant’s mean time course within the vection window (i.e. − 0.5 to + 4 s relative to vection onset) was extracted and exported to LORETA. In the incoherent trials there was no vection onset and thus no clear window of data to extract. In this case, each participant’s mean time course in a comparable window (i.e. 0.5 to + 4 s) was extracted and exported to LORETA, using their respective mean vection onset latency from the coherent condition as a marker (i.e. time = 0). A transformation matrix based on the coordinates of the electrode positions was created and applied to the coherent and incoherent data. To test for significant effects, paired samples *t* tests were conducted between the LORETA transformed coherent and incoherent conditions at each time point, for both left and right handers.

### Statistics

#### Behavioural data

For both the coherent and incoherent conditions the following behavioural data were obtained for every trial: (1) vection presence, i.e. if vection was reported in a given trial, (2) onset latency, i.e. the time between motion onset and vection onset, (3) duration, i.e. how long the period of vection lasted and (4) vection strength, i.e. subjective rating of how strong the vection experience was from 0 = ‘no vection’ to 10 = ‘I felt I was really moving’. To verify that CW and CCW trials could be appropriately collapsed, Wilcoxon signed-rank tests were conducted to compare each behavioural measure during CW versus CCW stimulation, within the coherent and incoherent conditions, for left and right handers, respectively. As no effect of stimulation direction was observed on any behavioural measure (Wilcoxon signed-rank tests, all *p* > 0.05, Bonferroni corrected), CW and CCW stimulus directions were collapsed within the coherent and incoherent conditions, for all subsequent analyses. Potential differences between left and right handers on behavioural measures were assessed using the non-parametric Wilcoxon rank sum test within both the coherent and incoherent conditions. Effect sizes were calculated as *r *= *Z*/√ (number of observations). An additional correlation analysis was conducted to investigate potential habituation of vection strength over the course of the experiment, for both the coherent and incoherent conditions. The vection strength scores for each subject were first normalized by subtracting their respective median score over all trials. The data were pooled for left and right handers and a Spearman’s rank-order correlation was conducted to compare normalized vection strength over trials.

#### FFT analysis

Potential frequency band differences between the coherent and incoherent conditions were examined by means of nonparametric permutation testing, for left and right handers, respectively. The test examined whether there was a significant difference in power, at the individually defined peak, in the coherent versus incoherent conditions at each electrode, for each participant. Specifically, the peak values in the coherent and incoherent conditions were shuffled for each participant over 1000 iterations. Significance values were obtained by comparing the observed group test statistic with the null distribution by converting the observed test statistic into a standard *Z* value and then converting it to a *p* value [40]. A cluster-based permutation test, following the procedure outlined in [41], was then implemented as a way to take the problem of multiple comparisons and data dependency into account in the statistical testing procedure. All electrodes for which the permuted *Z* value exceeded an a priori threshold (*p* < 0.05) were clustered on the basis of spatial adjacency. The data were then randomized over 1000 iterations to obtain a null distribution of the largest cluster *Z* values. This distribution was then compared against the observed cluster-level statistic (defined as the sum of the *Z* values within the cluster) and a *p* value calculated.

#### TF analysis

Potential differences in the alpha band activity around vection onset and during the course of ongoing vection were also assessed using nonparametric permutation testing, for left and right handers within their respective ROIs. In this instance the test examined whether there was a significant difference between the averaged baseline window (i.e. − 1.5 s to − 0.5 s relative to vection onset) and each time point in the vection window (i.e. − 0.5 s to + 4 s relative to vection onset). In detail, the alpha band value at baseline and in the vection window were shuffled for all time points, for each participant over 1000 iterations. Significance values were obtained as outlined above before a temporal cluster-based permutation test was conducted. In this instance, clusters were defined as having a minimum of 20 sequential time points for which the permuted *Z* value exceeded an a priori threshold (*p* < 0.05). The data were then randomized over 1000 iterations to obtain a null distribution of both the largest positive and negative cluster *Z* values. These distributions were then compared against their respective observed cluster-level statistic (defined as the sum of the *Z* values within the cluster) and *p* values calculated. All statistical analyses were conducted in MATLAB using custom scripts.

## Results

### Behavioural data

#### Vection presence

For each trial, the presence or absence of vection was recorded. These data were then summarized as the overall percentage of trials in which vection was reported as present for both the coherent and incoherent conditions. In the coherent condition, the median vection presence was 96% for left handers and 94% for right handers, with no significant difference (*Z* = − 0.66, *p* = 0.51, *r* = 0.13). In the incoherent condition median vection presence reported was 4% for left handers and 8% for right handers, a difference which did not reach significance (*Z* = − 0.92, *p* = 0.36, *r* = − 0.18). Note that the vast difference in the percentage of trials in which vection was reported present between the coherent and incoherent conditions precludes meaningful statistical comparison between the two conditions.

#### Onset latency

In the coherent condition the median onset latency was 5.96 s for left handers and 5.61 s for right handers, with no significant difference (*Z* = − 0.27, *p* = 0.79, *r* = − -0.05). In the incoherent condition, the left handers had a median onset latency of 10.72 s, with the right handers having a median of 12.66 s, a difference which did not reach significance (*Z* = − 1.12, *p* = 0.26, *r* = − 0.22).

#### Duration

In the coherent condition, a median vection duration of 12.48 s and 13.16 s was reported for left and right handers, respectively, with no significant difference between the groups (*Z* = 0.11, *p* = 0.91, *r* = 0.02). In the coherent condition the left handers had a median duration of 5.07 s, with the right handers having a median duration of 3.86 s, a difference which did not reach statistical significance (*Z* = 0.37, *p* = 0.71, *r* = 0.07).

#### Vection strength

The median vection strength reported in the coherent condition was 5.00 for left handers and 5.50 for right handers, with no significant statistical difference between the two groups (*Z* = − 1.25, *p* = 0.21, *r* = − 0.25). In the incoherent condition the median vection strength was 0 for both left and right handers, with no significant difference between the two groups (*Z* = − 0.06, *p* = 0.96, *r* = − 0.01). The Spearman’s rank-order correlation revealed a small negative correlation, with vection strength decreasing over trials for both the coherent (*r*_*s*_ = − 0.08, *p* < 0.001) and incoherent conditions (*r*_*s*_ = − 0.08, *p* < 0.001). This small negative correlation corresponds to a mean decrease of vection strength by 0.24 in the coherent condition and 0.35 in the incoherent condition, between the start and end of the experiment (mean of first 10 versus last 10 trials).

**Fig. 2:**
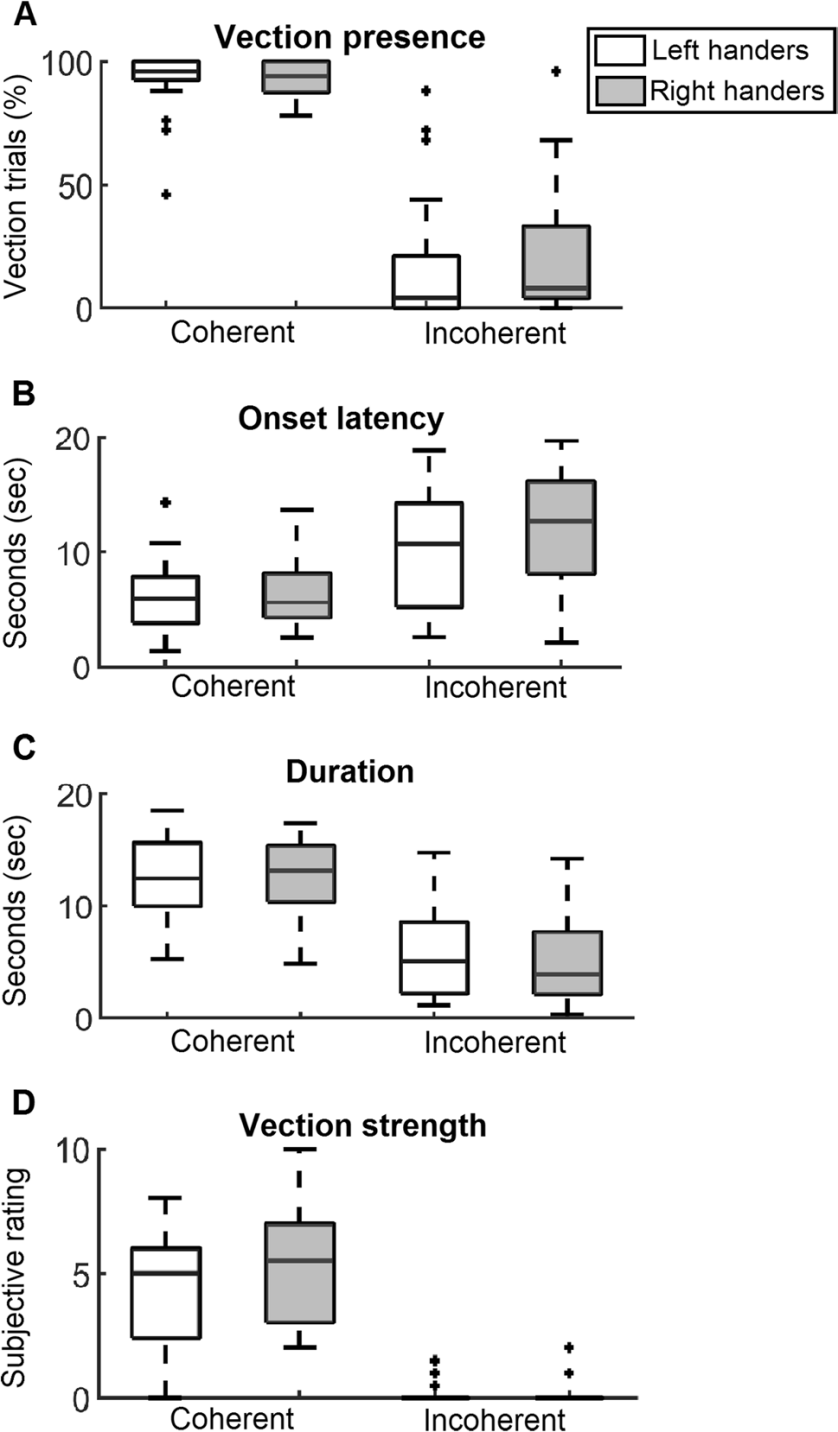
Behavioural data. **a** Vection presence, i.e. percentage of trials in which vection was reported as present. **b** Onset latency, i.e. time from motion onset to vection onset, in seconds. **c** Duration, i.e. length of vection period, in seconds. **d** Vection strength ratings on a scale of 0 (‘no vection’) to 10 (‘I felt I was really moving’). Each panel presents a boxplot with the median group value for the coherent and incoherent conditions, for both left (white) and right (grey) handers. The box around the median represents the 25th and 75th percentile, with the whiskers extending to the most extreme scores. Crosses represent outliers, calculated as values greater than *q*3 + *w *× (*q*3 − *q*1) or less than *q*1 − *w* ×(*q*3 − *q*1), where *w *is the maximum whisker length and *q*1 and *q*3 are the 25th and 75th percentiles, respectively

In summary, in both the coherent and incoherent conditions no statistically significant differences were observed between left and right handers on measures of vection presence, onset latency, duration and vection strength (Fig. 2).

### EEG data

#### Coherent versus incoherent visual stimulation (FFT analysis)

Potential differences in theta, alpha and beta activity between the coherent and incoherent conditions were examined by statistically comparing the obtained power between both conditions, at each electrode, for left and right handers, respectively. Significant differences between the coherent and incoherent conditions were observed only in the alpha band.

**Fig. 3:**
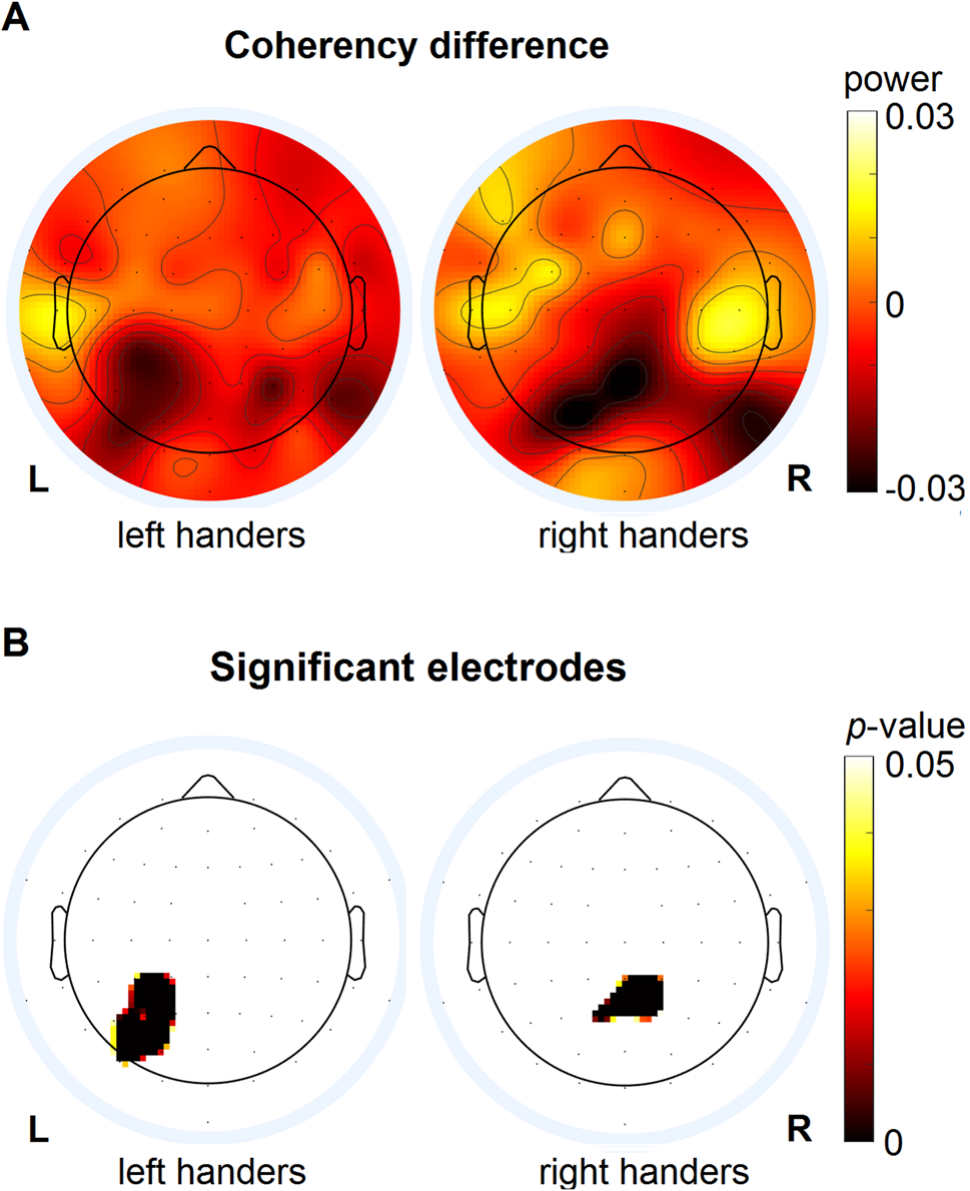
Coherent versus incoherent visual stimulation (FFT analysis)*.* **a** Coherency difference: Topographies showing group–level coherency difference, i.e. coherent condition minus incoherent condition, in alpha power for left and right handers, respectively. **b** Significant electrodes: Topographies showing the largest cluster of electrodes in which alpha power differed significantly between the coherent and incoherent conditions, i.e. *p* < 0.05 after cluster-based permutation testing, for both left and right handers. Both groups showed a decrease in alpha power in the coherent relative to incoherent condition. For left handers (bottom left) the significant reduction in alpha power in the coherent condition was visible at a left centro-parietal region including electrodes CP1, P3, CP3, P1, P5, PO7, and PO3, while right handers (bottom right) showed the effect at a midline centro-parietal region including electrodes Pz, CP2, P1, P2 and CPz

The mean difference in alpha power between the coherent and incoherent conditions, i.e. coherent minus incoherent, for both left and right handers, is illustrated in Fig. 3a. After cluster-based permutation testing to deal with the issue of multiple comparisons, significant differences between the coherent and incoherent conditions were observed for both left and right handers (Fig. 3b). For left handers, there was a significant difference between coherent and incoherent conditions at a left centro-parietal cluster, including electrodes CP1 P3, CP3, P1, P5, PO7 and PO3 (*p* = 0.0004). Right handers showed a significant difference between the coherent and incoherent conditions at a midline centro-parietal cluster, including electrodes Pz, CP2, P1, P2 and CPz (*p* = 0.02). In both instances, the statistical differences were driven by a *decrease* in alpha power in the coherent condition relative to the incoherent condition. Notably, the behavioural data (Fig. 2) descriptively show that the experience of vection was more intense in the coherent than incoherent condition, i.e. present on more trials, shorter onset latency, longer duration and stronger subjective experience. Indeed, on a group level vection was reported as occurring on as little as 4% and 8% of trials for left and right handers, respectively, in the incoherent condition, meaning this condition was less vection compatible than the coherent condition. Combined with the present results, this would indicate that alpha power decreased during a vection compatible stimulus, i.e. coherent condition, relative to a comparable stimulus which was less likely to induce vection, i.e. incoherent condition. In other words, the decrease in alpha power appears to occur in the presence of vection, with the effect being observed at distinct electrode clusters for both left and right handers.

#### Temporal dynamics of vection (TF analysis)

**Fig. 4:**
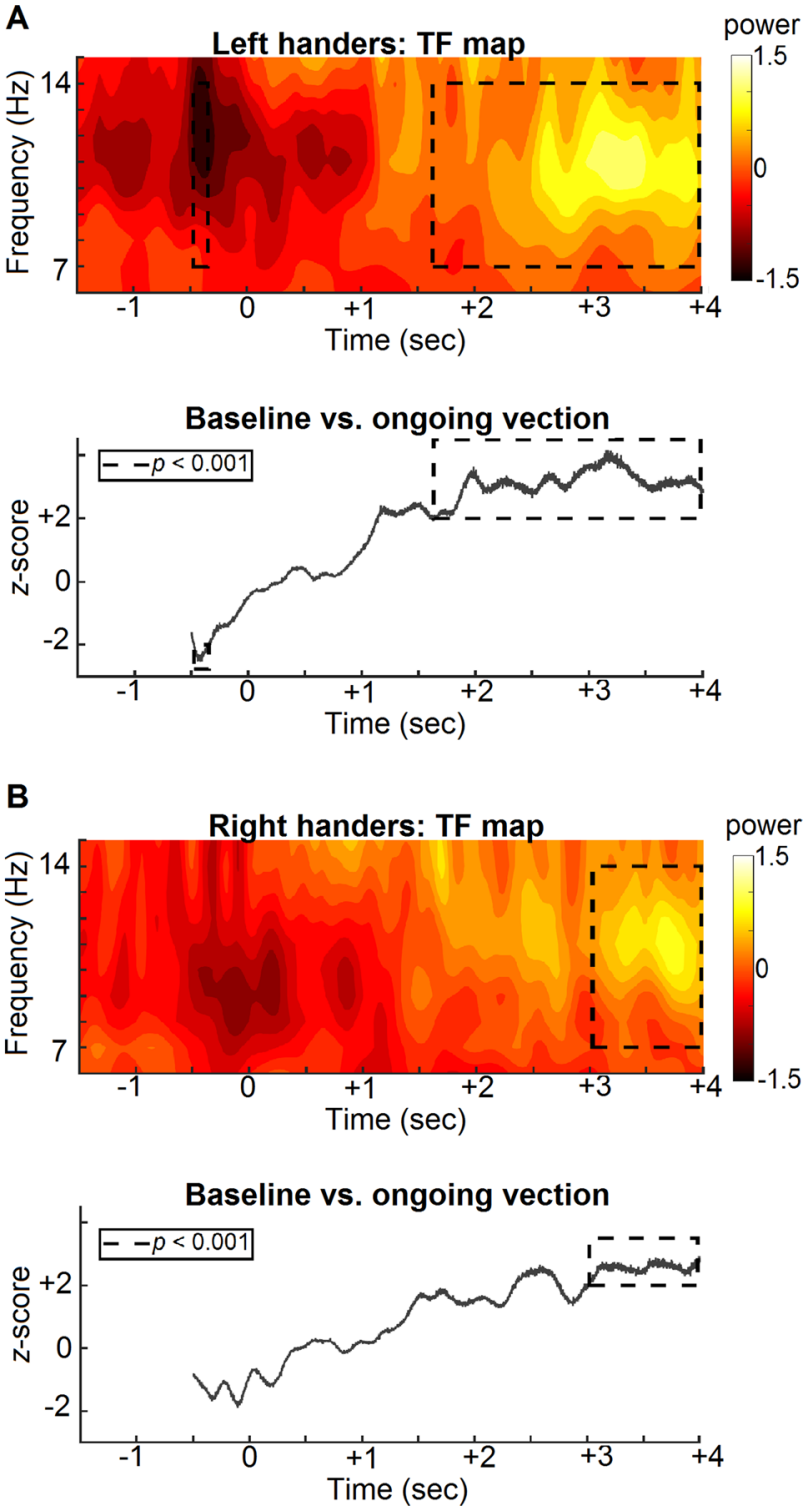
Temporal dynamics of vection (TF analysis) at handedness-specific ROIs for left handers (**a**) and right handers (**b**). *Time frequency (TF) maps* show changes in alpha power time locked to vection onset, i.e. time 0. *Baseline vs. ongoing vection maps* show the *z* score difference in alpha power between the baseline window, i.e. − 1.5 s to − 0.5 s, and the ongoing vection window, i.e. − 0.5 s to + 4 s, at each time point. Regions surrounded by black dotted lines are significant at *p* < 0.001 after cluster-based permutation testing. Both left and right handers show similar changes in alpha power over the course of vection, with a decrease in power being observed around the time of vection onset (significant for left handers only) and an increase in power being observed during ongoing vection

The temporal dynamics of alpha band activity during the course of vection were examined by statistically comparing the alpha power observed in the averaged baseline window, i.e. − 1.5 s to − 0.5 s relative to vection onset, with the alpha power observed at each time point in the vection window, i.e. − 0.5 s to + 4 s relative to vection onset (Fig. 4). This analysis was conducted on coherent trials only, with separate ROIs being used for left and right handers, i.e. ROIs were defined as the clusters of electrodes which differed significantly between the coherent and incoherent conditions. For left handers, cluster-based permutation testing revealed two periods of time in which alpha power differed significantly between the baseline and vection windows. Firstly, relative to baseline there was a significant *decrease* in alpha band power prior to vection onset (*p* < 0.001, from − 0.47 to − 0.34 s relative to vection onset). Secondly, relative to baseline there was a significant *increase* in alpha power during ongoing vection (*p* < 0.001, from + 1.63 to + 3.98 s relative to vection onset). For right handers a significant increase in alpha power, relative to baseline, was observed during ongoing vection (*p* < 0.001, from + 3.02 to + 3.98 s relative to vection onset). Notably, both groups demonstrated earlier significant increases in alpha band power during ongoing vection (left handers: from + 1.13 to + 1.62 s; right handers: from + 2.36 to + 2.73 s). However, as the cluster-based permutation testing was conducted on the maximal positive and negative clusters, these remain uncorrected for multiple comparisons. In summary, these results show that alpha power decreased prior to vection onset (significant for left handers only) and increased during ongoing vection.

#### Source localization

For both left and right handers, comparison of the LORETA transformed coherent and incoherent conditions returned non-significant results (*p* > 0.05). Although non-significant, the maximal differences between coherent and incoherent conditions included a number of regions associated with the vestibular network [21, 28]. For left handers, the maximum differences were observed at left postcentral gyrus, precentral gyrus and inferior parietal lobule. For right handers, the maximal differences were observed at left inferior parietal lobule, postcentral gyrus, inferior frontal gyrus, precuneus, insula and right paracentral lobule.

## Discussion

The present study examined the behavioural characteristics and neural correlates of visually induced vection in left and right handed participants. Our results found no difference between left and right handers on behavioural measures of vection, i.e. presence, onset, duration and subjective strength, in either the coherent vection–compatible condition or the incoherent control condition. Additionally, we found no evidence amongst left and right handers to suggest that these behavioural measures of vection are modulated by stimulus direction (i.e. CW versus CCW). The results of the FFT analysis demonstrated a significant decrease in alpha power in the coherent relative to incoherent conditions, for both left and right handers. Notably, this difference was observed in different topographical regions, with left handers demonstrating a decrease in left centro-parietal electrodes and right handers showing a decrease at midline centro-parietal electrodes (Fig. 3). Neither left nor right handers exhibited significant differences in theta or beta band activity between the two conditions. In order to further investigate the relationship between alpha power and vection, a time–frequency analysis, time locked to vection onset was conducted. The results of this analysis found significant changes in alpha power during the course of vection, with similar patterns being observed for left and right handers. The present study is the first comparison of the behavioural characteristics and neural patterns of roll vection across left and right handers. The implications of our study findings for our understanding of visual self-motion are discussed below.

### Behavioural characteristics of roll vection are independent of handedness

In the present study, left and right handers demonstrated remarkably similar vection characteristics with almost identical scores on measures of vection presence, onset latency, duration and subjective strength, for both coherent and incoherent conditions. To some extent this finding contradicts the recent observation that right handers display quicker perceptual transitions from world- to self-motion (i.e. vection onset latency), compared to left handers [42]. One possible explanation for the discrepancy between these findings is differences in the employed visual motion stimuli and the vection they induced. In the previous study, the optokinetic stimuli consisted of a drum marked with vertical black and white lines, which rotated around the participant in the earth-vertical axis, inducing circular vection. In contrast, the visual motion stimuli used in the present study consisted of white dots on a black background, rotating along the roll axis, inducing the perception of roll vection. Notably, the onset latencies in the present study were considerably shorter than those reported in the previous study [42], which could be indicative of a more intense vection experience resulting in less subjective variation on behavioural measures across participants. Although literature on the topic is sparse, there is some evidence showing differences in onset latency [1] and subjective intensity [4] across different types of vection, i.e. along different planes/axes. Indeed, different types of motion stimuli have also been found to show different patterns of neural activation both in the absence [43] and presence [4] of vection. Such findings make it difficult to compare and interpret the relevance of differences in onset latency findings between this and the previous study. A small negative correlation, across both left and right handers, revealed that vection strength decreased very slightly over the course of the experiment. Crucially, this small decrease in perceived vection strength was comparable across the coherent and incoherent conditions, suggesting that both conditions were comparably affected by slight vection habituation over time. In summary, the behavioural data indicate that left and right handers experience roll vection in a similar manner.

### Alpha power is decreased during vection induced by visual motion stimulation

In our study, both left and right handers exhibited decreases in alpha power whilst viewing a vection–compatible visual motion stimulus, in contrast to a control stimulus. Determining whether changes in activation patterns reflect visual motion stimulation in general, differences between experimental and control conditions and/or vection itself is experimentally challenging. For example, studies which contrast vection-inducing visual motion stimuli with static control stimuli, e.g. [4, 44], likely include activation changes that reflect the difference between stationary and moving stimuli, irrespective of whether or not vection was present. Also, contrasts between coherent and incoherent/random motion stimuli may result in activation changes which are attributable to physical stimulus differences rather than vection per se [2, 45]. As such, it is apt to examine the extent to which the decrease in alpha power, observed in the present study, can be attributed to vection.

Firstly, the behavioural results clearly demonstrate that on the group level, vection was almost always present in the coherent visual motion condition (i.e. on 96% and 94% of trials for left and right handers, respectively) but rarely, if ever, present in the incoherent visual motion condition (i.e. on 4% and 8% of trials for left and right handers, respectively). This demonstrates that the observed decrease in alpha power is associated with the presence of vection. Secondly, the incoherent visual control stimulus was designed such its physical stimulus properties matched those of the coherent stimulus as closely as possible. Although the incoherent stimulus contained additional local sinusoidal motion, both stimuli presented the same average global velocity. Physical stimulus differences would have likely produced differences in occipital activations between the two conditions, reflecting early visual processing. The absence of such occipital activations in our results suggests that our findings are not attributable to differences between the employed stimuli. Furthermore, this also indicates that the observed differences contain limited, if any, activity related to the processing of visual motion stimulation rather than vection per se. Lastly, subsequent time–frequency analysis of vection-present coherent trials, showed changes in alpha power which were time locked to vection onset, for both left and right handers. This provides further evidence that the changes in alpha power observed in this study are related to vection, rather than general visual motion stimulation.

### Left and right handers show differences and similarities in vection related alpha power changes

A key finding in this study is that the decrease in alpha power that results from exposure to a vection compatible stimulus is observed at different topographical regions for left and right handers. Left handers exhibited a lateralized response to the vection stimulus over a left centro-parietal region. In contrast, right handers showed a bilateral response in a midline centro-parietal area (Fig. 3). Multiple comparisons can be drawn between these findings and the existing literature. First, although non-significant, the source localization results suggest that the vection-compatible stimulus, which results in different activity patterns for left and right handers, and the incoherent stimulus are maximally different at regions within the vestibular network. Although further research is required to substantiate this finding, it is congruent with the established idea that visually-induced vection relies on visual–vestibular interaction [2]. Secondly, in contrast to previous fMRI and PET studies [2–5], which demonstrate vection-related activation of visual cortex and concurrent deactivation of PIVC, the present study found that vection is associated with activity changes at centro-parietal regions. This discrepancy could be explained by a number of factors including our use of a novel control stimulus which controlled for low-level visual properties and average global motion, and also to differences in the type of activity and spatial resolution measured by EEG, fMRI and PET. Lastly, the observation that left and right handers show vection-related alpha band changes at different topographical regions is congruent with fMRI and PET studies demonstrating handedness-dependent vestibular thalamo-cortical dominance [17–28]. Notably, while such studies show vestibular dominance in the right hemisphere in right handers and in the left hemisphere for left handers, the present study found a bilateral activation for right handers and a left-lateralized activation for left handers.

Given the topographical differences in vection-related activity for left and right handers, subsequent time–frequency analyses were conducted separately for both groups in order to maximize our ability to examine the neural correlates of vection within each group. Despite the topographical differences in where vection-related activity was observed, both left and right handers exhibited a similar pattern of alpha band changes over the course of vection. Both groups showed decreased alpha power around vection onset (significant only for left handers) and increased alpha power during ongoing vection (Fig. 4). These findings not only compliment those of a recent EEG study examining the neural correlates of vection in right-handed participants [31], but also extend the findings to left handers. Notably, left handers appear to show increased alpha power during vection much earlier than right handers in our study. However, as both groups demonstrate the same trend of increasing alpha power during ongoing vection and that the measures of alpha power come from different topographical regions, we are hesitant to label this temporal discrepancy as an effect of handedness. The observation that left and right handers show similar alpha patterns during the course of vection matches well with our observation of comparable behavioural characteristics between the two groups and suggests that vection and its respective processes are consistent across handedness, despite those same processes being observed at different topographical regions in a handedness-dependent manner.

### A role for alpha oscillations in visually-induced vection

This study made two key observations about alpha oscillations in relation to vection: (1) a decrease in alpha power is observed during exposure to a vection-compatible stimulus, relative to a matched control and (2) both decreases and increases in alpha power are observed during the course of vection. Beginning with the former, the decrease in alpha power during exposure to a vection compatible stimulus is consistent with desynchronization and a release from inhibition [46], correlating with excited neural structures or activated cortical areas [47]. This would suggest that exposure to a vection compatible stimulus induces increased activity in centro-parietal regions, with the effect being (midline) bilateral for right handers and left-lateralized for left handers. Evidence exploring the relationship between alpha oscillations and activations observed in fMRI and PET imaging suggests that alpha power is negatively correlated with the blood-oxygen-level-dependent (BOLD) signal [48] and also with regional cerebral blood flow (rCBF) in primary and association visual cortex [49]. Although the origins of the alpha oscillations observed in this study are unclear, the inverse relationship between alpha power and cortical activation suggests that the observed decrease in alpha power in the present study maps well with findings of increased activation of parieto-occipital regions in previous vection studies [2, 4]. Such previous studies employed only right handed participants and therefore, it is unclear whether spatial differences between left and right handers similar to those observed in this study, would also present themselves in parieto-occipital activations in PET and fMRI studies.

Although our findings indicate a clear link between alpha oscillations and vection, the exact nature of this relationship requires further investigation. The first possibility is that the decrease in alpha power reflects an increase in cortical activation which is compatible with an inhibitory visual–vestibular interaction. This increased activation could operate by amplifying visual signals over vestibular signals such that the visual dominates or by triggering inhibition of vestibular cortex itself. A second possibility is that decreased alpha power reflects an inhibitory visual–visual interaction, with the increased activation either amplifying visual signals consistent with self-motion over those consistent with object–motion or even triggering inhibition of visual object–motion processing.

A third possibility is that the decreased alpha and increased activation reflect additional attentional processes involved in vection perception. It is possible that the sensation of self-motion and/or the sensory mismatch involved in vection perception might require or induce additional attentional processes which involve increased cortical activation. For example, alpha desynchronization has also been observed during turning movements in virtual reality environments, with stronger decreases occurring when there is incongruency between sensory modalities and thus, increased demands on visuospatial attention [50]. Furthermore, the observed decrease in alpha power in the present study could also reflect top-down attentional control. Vection requires that visual motion is interpreted as resulting from self-motion rather than environmental motion. This erroneous interpretation can be explained by our a priori anticipation that the external world is stable, making it more probable that motion cues are attributable to self-motion [1]. In terms of visual processing, anticipatory alpha band modulations have been implicated in the top-down allocation of selective visuospatial attention [51]. Further, the amplitude of alpha desynchronization has been shown to follow the time course of temporal expectation, suggesting that alpha oscillations have a role in the regulation of cortical excitability as a function of anticipatory visuospatial attention and may act as a mechanism for biasing perception [52]. Indeed, in addition to the decreases in alpha power during exposure to a vection compatible stimulus, the time–frequency analyses also revealed a decrease in alpha power beginning prior to vection onset. In this context, this finding could be interpreted to reflect top-down attentional control and anticipation of vection. This decrease in alpha power around the time of vection onset is also consistent with the literature on bistable perception, in which decreasing alpha power is thought to reflect destabilization of the current percept, with decreases beyond a given threshold resulting in the development of the alternative percept [32, 34].

During ongoing vection, we observed an increase in alpha power, which has been shown to be associated with increased inhibition and reduced activation [46]. On one hand, this increase in alpha power during ongoing vection could be a result of object–motion compatible signals and/or vestibular signals (i.e. indicating a lack of self-motion) exerting a reciprocal inhibition on visual self-motion signals. The temporal characteristics of vection vary widely between participants and in order to ensure sufficient trials for analysis and that there was no contamination from changes in alpha power due to vection or stimulus offset, we selected a very limited time window for our analysis of ongoing vection. Unfortunately, this means that we do not know if alpha power continued to increase beyond our window of investigation and if that decrease would ultimately lead to the breakdown of vection. An alternative explanation for the increased alpha power is that while an initial decrease in alpha and an associated increase in activity, is necessary for one perceptual interpretation (i.e. self-motion) to become dominant, the dominant percept can be sustained without continuing increased activation, thus allowing a shift to a less active baseline state with greater alpha power. We propose that future studies combining EEG and non-invasive brain stimulation techniques (e.g. TMS) or positron emission tomography (PET) are necessary to disentangle the role of alpha power increases and decreases and to establish a causal link between alpha oscillations and vection.

## Conclusion

This study examined the behavioural characteristics and neural correlates of roll vection in left and right handers. We found that vection-related alpha power changes occur at different topographical regions for left and right handers. Despite these spatial differences, both left and right handers exhibit similar behavioural characteristics and patterns of alpha band changes during ongoing vection.

## Availability of data/materials

Data are not publicly available due to the constraints of the ethical approval for the study. Example code is available from the corresponding author on reasonable request.
